# Caregivers’ perspectives of early developmental tele-assessments in challenging circumstances

**DOI:** 10.4102/sajcd.v71i1.1037

**Published:** 2024-07-23

**Authors:** Maria N. du Toit, Renata Eccles, Kailin Westwood, Marien A. Graham, Jeannie van der Linde

**Affiliations:** 1 Department of Speech-Language Pathology and Audiology, Faculty of Humanities, University of Pretoria, Pretoria, South Africa; 2 Department of Science, Mathematics and Technology Education, Faculty of Education, University of Pretoria, Pretoria, South Africa; 3 Department of Early Childhood Education, Faculty of Education, University of Pretoria, Pretoria, South Africa

**Keywords:** caregiver interview-based assessment, early identification, caregiver report, tele-assessment, COVID-19

## Abstract

**Background:**

Outbreaks, such as the COVID-19 pandemic in 2020, exacerbate barriers to accessing early childhood developmental care. Tele-assessment may serve as an innovative approach to developmental monitoring to overcome service delivery amidst challenging circumstances. It is vital to collect caregivers’ perspectives of this potential service delivery method to inform clinical decision making.

**Objectives:**

This study aimed to determine caregivers’ perspectives of interview-based early developmental tele-assessment in a South African context.

**Method:**

Thirty caregivers of children (aged birth – 36 months) completed a caregiver-report developmental assessment via a telecommunications platform, as well as an online questionnaire probing their perspectives on the tele-assessment.

**Results:**

Most participants (96.7%, *n* = 29 out of 30) rated their overall experience of the tele-assessment as positive; however, 53.8% (*n* = 14 out of 26 that answered the question) indicated that they would additionally still prefer in-person assessment.

**Conclusion:**

Tele-assessment appears to be a viable approach for caregivers to access developmental care during circumstances such as COVID-19.

**Contribution:**

This study provided valuable insight into a novel approach using interview-based early developmental tele-assessment and the perspectives of caregivers thereof.

## Introduction

In low- and middle-income countries (LMICs), such as South Africa, environmental risk factors, including poverty and malnutrition, as well as biological risk factors such as exposure to human immunodeficiency virus (HIV) among others, impact the development of children younger than 5 years of age (Richter et al., 2017). Developmental monitoring for early identification of delays is vital for families of children exposed to these risks. Access to consistent early identification of these delays is, however, challenging in many countries including South Africa. Although South Africa was recently reclassified as an upper-middle-income country (UMIC), the country still has many low-income areas that are faced with disparities in socio-economic status and service delivery (World Bank, 2020). An additional challenge faced by children of all socio-economic groups in need of developmental monitoring is the shortage of healthcare professionals, leading to decreased access to healthcare services (Maphumulo & Bhengu, 2019). While community health professionals, such as nurses, at primary health care clinics (PHCs) can conduct developmental screening, only 40% of PHCs have employed staff with the necessary training to administer developmental screening and assessment (Van der Merwe et al., 2017). Thus, children are not receiving regular and consistent developmental monitoring (Khoza-Shangase & Mophosho, 2018).

Access to developmental services is further reduced in times of crisis, as with the outbreak of the coronavirus disease (COVID-19) pandemic in 2020. The global response by health authorities was largely focused on the prevention of infection by placing a large portion of the global population under strict regulations to enforce social distancing guidelines (Salawu et al., 2020). South Africa, specifically, was placed under a strict lockdown in March 2020, which is now considered to have been one of the most restrictive lockdowns in the world. The population was only allowed to leave their homes for essential grocery shopping and medical reasons – and many citizens had to work remotely from home. Other challenges that also frequently arise and affect in-person service delivery include protest action by healthcare workers and/or public transport services because of wage disputes and water shortages forcing healthcare facilities to keep their doors closed (Maphumulo & Bhengu, 2019; Masiya et al., 2019). During challenging circumstances, access to developmental healthcare, such as routine child wellness visits, is further limited. Immunisation appointments, which often occur during child wellness visits, are considered effective opportunities for child healthcare as they can serve as proxies for children’s access to other important services including developmental monitoring (Lake et al., 2019). Children who would have been provided the opportunity for developmental monitoring during child wellness visits missed appointments because of strict lockdown regulations as well as caregivers’ concerns about exposing their children to COVID-19 (Santoli et al., 2020). Although citizens could leave their homes for medical reasons, many did not unless it was considered a medical emergency (Stiegler & Bouchard, 2020). Consequently, at-risk children experienced further reduced access to developmental healthcare.

In response to the physical restrictions to healthcare access and service delivery during COVID-19, alternative solutions for continued service delivery were considered. Allied healthcare professionals predominantly turned to tele-practice as an alternative means of service delivery (Hollander & Carr, 2020; Tohidast et al., 2020). Tele-practice is defined as the use of telecommunication technologies to deliver services, such as health promotion, assessment or intervention, to those who are in a different physical location than the service provider (Cohn & Cason, 2019). This form of service delivery has been endorsed by the American Speech-Language-Hearing Association (ASHA) as an effective and viable means of service delivery (Lowman & Kleinert, 2017). In South Africa, tele-practice in the form of health promotion text messaging is garnering attention in the realm of developmental care through mHealth initiatives such as MomConnect. This initiative supports pregnant women and parents with information on health and developmental milestones (Barron et al., 2016). While there has been a growth in the evidence supporting the effectiveness of mHealth and tele-practice, especially in response to challenging circumstances, few studies have explored the perspectives of parents or caregivers on this mode of service delivery. Furthermore, contextual factors, such as socio-economic status and family characteristics, that may influence caregivers’ perspectives (Ayob et al., 2021; Draper et al., 2023) should also be considered.

Certain barriers may influence the effectiveness of tele-practice. Barriers include data costs, trouble establishing rapport with clients, lack of access to necessary technologies and ethical issues including confidentiality, payment and clinician’s competency to provide tele-practice (Fong et al., 2021; Tohidast et al., 2020). In recent years, however, allied healthcare professionals in LMICs, such as India, have used tele-practice to increase access to clients who reside in remote locations (Mohan et al., 2017). Digital health solutions are rapidly growing, both in number and capability – despite this, confidence in these solutions among stakeholders – including clinicians and patients – remains low (Mathews et al., 2019). This is because of the current lack of guidelines or frameworks assisting clinicians to identify validated digital health solutions. In such a fast-advancing field where it is challenging to determine what is best practice, rolling out a digital health solution on a larger scale becomes an issue.

The American National Association of School Psychologists reported that tele-assessment is the least researched area of tele-practice and issues subsequently arose when tele-assessment became more necessary in response to COVID-19 (Krach et al., 2020). ‘Adapted tele-assessment’, or the use of assessment designed for traditional face-to-face assessments in a tele-assessment context, was suggested to be used cautiously in response to the COVID-19 crisis and until more research was available (Krach et al., 2020). There is a need to investigate tele-assessment in far more studies moving forward, as well as a means to investigate its viability. In order to facilitate this potential body of research, it is important to consider key stakeholders, such as caregivers, and their opinions regarding tele-assessment.

There is an expanding body of research in South Africa supporting caregiver report as a means of developmental surveillance and assessment (Maleka et al., 2016; Van der Linde et al., 2016; Van der Merwe et al., 2017), as it is a time-efficient, accurate and cost-effective means of assessment (Arciuli et al., 2013; Bornstein et al., 2018; Crais, 2011; Engle et al., 2011) within all contexts including multilingual populations (Guiberson et al., 2011). Use of caregiver report to obtain assessment information is often also considered a more feasible means than clinician-administered assessments (McLeod et al., 2017), as caregivers can provide accurate judgements of their children’s functional abilities. Caregivers are most familiar with how their children participate in daily routines, which is a good measure of developmental ability (Balton et al., 2019; Romski et al., 2018). While the use of caregiver-report tools such as the Vineland-3 is supported in research for use during in-person interviews for the purpose of describing the developmental characteristics of a population (De Beer et al., 2020), the application of such a tool via tele-practice has not yet been explored in South Africa. The current research on caregiver-report and tele-practice in South Africa is geared more towards screening and screening tools, rather than assessment.

The Parents’ Evaluation of Developmental Status (PEDS) and the PEDS: Developmental Milestones (PEDS:DM), known as the PEDS tools combined, is a screening tool that has recently been investigated as a digital health solution in South Africa (Du Toit et al., 2021). The caregiver-report screening tool, administered on a smartphone application, has shown great potential in the screening of children in the PHC context (Van der Merwe et al., 2019). As caregiver-report via mobile health works well in terms of screening, the progression to comprehensive developmental assessment should be explored. Caregiver-report interviews via tele-assessment may provide a means to overcome barriers to service delivery. A potential benefit is that children do not need to be physically present and thus, in the context of a global crisis, the health professional, caregivers and children remain safe (Tohidast et al., 2020). Tele-assessment may provide an innovative approach to developmental monitoring. This study predominantly aimed to explore caregivers’ perspectives regarding early developmental tele-assessments. To better understand their perspectives, we investigated if any demographic aspects were associated with their perspectives as possible associated factors. A sub-aim of the study was to investigate possible associated factors between caregivers’ demographic characteristics and their perspectives.

## Research methods and design

### Research design

An embedded mixed-method survey design was used to describe the outcomes of a caregiver-report developmental assessment administered by a speech-language pathologist via tele-practice and the perspectives of caregivers on the tele-assessment process. Quantitative data were collected through surveys administered to a sample of participants, focusing on demographic information and key variables related to the research questions. Concurrently, qualitative data were gathered through in-depth interviews with a subset of participants, allowing for a deeper exploration of their experiences and perspectives (Creswell, 2014). The qualitative research design was based on grounded theory as the study findings were based on data generated from participants who prior to completing a questionnaire regarding their perspectives of tele-assessment, had participated in a tele-assessment of their young child (Creswell & Poth, 2016).

### Participants

Non-probability, snowball sampling was employed to recruit 30 caregivers of infants and young children between the ages of birth and 36 months. Snowball sampling is used when the population, who have a set of unique characteristics, is not easily accessible (Naderifar et al., 2017). In the context of this study, the population was less accessible because of the COVID-19 pandemic. The participants were thus recruited via word of mouth – a message detailing the study, and its purpose was circulated to people known by the researcher, who sent the message to others who met eligibility criteria thereafter. To meet the eligibility criteria, caregivers had to: (1) be the primary caregiver of an infant or child between birth and 36 months, (2) be over 18 years of age, (3) understand conversational English to answer questions from the assessment measure, (4) have access to the internet, as well as a working computer or laptop or phone with a microphone and (5) have an active email address to use the telecommunications platform and to receive the link to the online questionnaire.

### Material

Three different measures were utilised for data collection: A researcher-developed demographic questionnaire to obtain the family’s demographic information, the Vineland 3 Adaptive Behavior Scales (the Vineland-3) Caregiver Interview (Sparrow et al., 2016) and an online researcher-developed questionnaire regarding caregiver perspectives of the developmental assessment format.

The questionnaire investigating perspectives took approximately 5–10 min to complete. The online questionnaire consisted of Likert-type that probed caregivers’ experience of the tele-assessment in terms of clarity, technical difficulties and the platform used. Open-ended questions further collected caregivers’ opinions on how natural the tele-assessment felt, the feasibility of the tele-assessment format and its applicability for future use.

The Vineland-3 (Sparrow et al., 2016) is a standardised, norm-referenced assessment tool used to measure adaptive behaviour of individuals from birth to age 90. The tool is widely used for assessment and diagnosis of developmental delays and disorders (Sparrow et al., 2016). The previous edition of the tool, the Vineland-2, has been used successfully in UMICs and LMICs, such as India and Pakistan (Rahman et al., 2016) and South Africa (Sipsma et al., 2013). The Vineland-3 has also recently been used successfully in South Africa (De Beer et al., 2020; Du Toit et al., 2021).

The Vineland-3 caregiver interview form involves the use of close-ended questions with suggested phrasing to elicit more elaborated responses from caregivers to ensure ease of understanding. The domains targeted by the tool in the age range of this study (birth to 36 months) include the developmental domains of receptive language, expressive language, personal adaptive behaviour, interpersonal relationships, play and leisure, coping skills and motor skills, in accordance with the guidelines of the tool (Sparrow et al., 2016).

An online questionnaire was developed to collect participants’ perspectives of the early developmental tele-assessment format. The Qualtrics software was used to create an online questionnaire, which consisted of Likert-type rating scales, ranging from strongly agree to strongly disagree, and open-ended questions. Open-ended questions were used to gather the caregivers’ opinions on the feeling of normality of the tele-assessment, its feasibility as a tele-assessment tool and its applicability for future use.

### Data collection procedures

The data collection took place as a single online session via Google Meet™, a secure end-to-end encrypted telecommunications platform. The online session was recorded and stored in an access-protected Google Drive folder. During the online session, the researcher-developed demographic questionnaire was completed first after which the Vineland-3 was administered via a caregiver interview.

After the interview, the online questionnaire was sent to each participant. The data collection process took between 40 and 90 min per participant, depending on the age of the participant’s child. After data were collected, the outcomes of each developmental assessment were calculated. The caregivers received feedback on their children’s results via email, and referrals were made if concerns were identified.

### Data analysis

The integration of quantitative and qualitative data occurred during the data analysis phase, where quantitative findings provided a foundational understanding, while qualitative insights enriched and contextualised the results. This embedded approach allowed us to triangulate data and provide a nuanced interpretation of the research outcomes (Creswell, 2014).

An a-priori power analysis was conducted using the G*Power software version 3.1.9.4 (Faul et al., 2007), and it was found that the minimum required sample size for a power of at least 0.8 was *n* = 29. The assessment results were processed according to proposed guidelines of the Vineland-3 scoring manual to determine whether children had a developmental delay or not (Sparrow et al., 2016). Raw scores were converted to v-scale scores using norm tables. The v-scale scores are norm-referenced scores with a mean of 15 and a standard deviation (SD) of three. The v-scale scores reflect how the raw scores for each subdomain compare with the norm sample of the age group. An adaptive behaviour composite (ABC) score is also calculated to describe the child’s overall level of adaptive functioning, based on the scores for the communication, daily living skills and socialisation domains. The ABC scores have a mean of 100 (SD = 15). These scores were used to determine the developmental domains in which delays were present.

Qualitative data were analysed using qualitative content analysis. Content analysis allows for data to be analysed qualitatively as well as enabling the data to be quantified. A descriptive approach was used to identify themes in the dataset and subsequently coded. Three major themes for each open-ended question were identified and coded: (1) Safety during challenging circumstances, (2) Convenience and (3) Practicality. Every participant’s answer to these questions was thus captured according to theme with the corresponding code in Excel. This allowed for the measurement of the frequency of each different theme or category (Vaismoradi et al., 2013).

The dataset was imported from Excel into the Statistical Package for Social Sciences (SPSS) version 26 to run descriptive and inferential statistics. Descriptive statistics including mean, median and standard deviation were computed to summarise the overall nature of the data (Leedy & Ormrod, 2014). The chi-square test of independence was used to establish whether two variables, number of children and order of children and their preference for tele-assessment were associated or not. Fisher’s exact test was used for a more exact representation of where there were non-random associations between two variables and to interpret *p*-values as recommended for smaller sample sizes. For correlations between a continuous variable, and a categorical (ordinal) variable, Spearman correlations were used to interpret whether any relationships between values were statistically significant. Where *p*-values were equal to or less than 0.05 (*p* ≤ 0.05), variables were significantly associated (chi-square/Fisher’s exact) or significantly correlated (Spearman correlation).

### Ethical considerations

Ethical clearance to conduct this study was obtained from the Research Ethics Committee of the Faculty of Health Sciences of the University of Pretoria (No. HUM020/1219).

Caregivers who met the inclusion criteria emailed signed informed consent to the researcher before data collection. The data obtained were securely stored electronically on the University of Pretoria data repository. All data were treated with confidentiality, with only one researcher having access to the participants’ personal information. The data will be kept for a minimum of 15 years according to the University of Pretoria’s guidelines for data storage.

## Results

### Participant characteristics

A total of 30 caregivers, who were all mothers, took part in the study (Table 1). The majority of participants were caregivers living in the province of Gauteng (*n* = 28), with two households recruited from the provinces of Mpumalanga and Western Cape giving a total sample size of 30. Of these participants, 63.3% (*n* = 19 out of 30) reported that they were the primary caregiver, while the other 36.7% (*n* = 11 out of 30) reported that both caregivers (mother and father) were the primary caregivers. There was a relatively equal distribution of child gender, with 60.0% (*n* = 18 out of 30) of the children being female. Additionally, these participants were of a higher socio-economic status, representing a small portion of the South African population. After the tele-assessment, two participants’ children were identified with a developmental delay (6.7%, *n* = 2 out of 30). One child presented with a delay in both the receptive and expressive language domains, while the second child presented with delays in the expressive language and coping skills domains.

**TABLE 1:** Participant characteristics (*N* = 30).

Characteristics	Categories	*n*	%
Age of the child (months)	0–6	5	16.7
	7–12	8	26.7
	13–18	6	20.0
	19–24	6	20.0
	25 or more	5	16.7
Gender of the child	Female	18	60.0
	Male	12	40.0
Respondent’s relation to the child	Mother of the child	30	100.0
Primary caregiver	Mother	19	63.3
	Both parents	11	36.7
Birth order of the child	1st	17	56.7
	2nd, 3rd or 4th	13	43.3

#### Caregivers’ experience of the tele-assessment format

Most caregivers perceived the tele-assessment as natural as if the assessment were conducted in person (80.0%, *n* = 24 out of 30). There was a significant moderate negative correlation (*r* = -0.403, *p* = 0.027) between the number of children a caregiver had and their perspectives of tele-assessment as being as natural as in person assessments. The lower the number of children, the more likely the mother was to select ‘neutral’ when indicating a preference between tele-assessment and in-person assessment. When asked to clarify why or why not the participant perceived the tele-assessment as natural, the themes that arose from the 25 responses provided were: (1) preference for conducting interviews in person (28%, *n* = 7 out of 25), (2) the participant felt that the online interview was as natural as if it were in person (56%, *n* = 14 out of 25) and (3) the participant was familiar with the platform or video conferencing (16%, *n* = 4 out of 25) (Table 2).

**TABLE 2:** Verbatim responses to the question: Clarify why or why not you perceived tele-assessment as natural as if in person.

Participant number	Responses
P1	‘There were some slight delays and it was sometimes more difficult to interpret subtle social cues.’
P5	‘I feel like there were a few background distractions on my side which wouldn’t have happened in person.’
P25	‘Because of the video it’s similar to meeting in person.’
P28	‘It was as natural as you can get for an interview via Google meets but total warmth of such an interview gets a little bit lost. But it’s on the other hand very convenient to do it this way.’
P30	‘Due to the nature of my work I have regular meetings via such a platform.’

**FIGURE 1:**
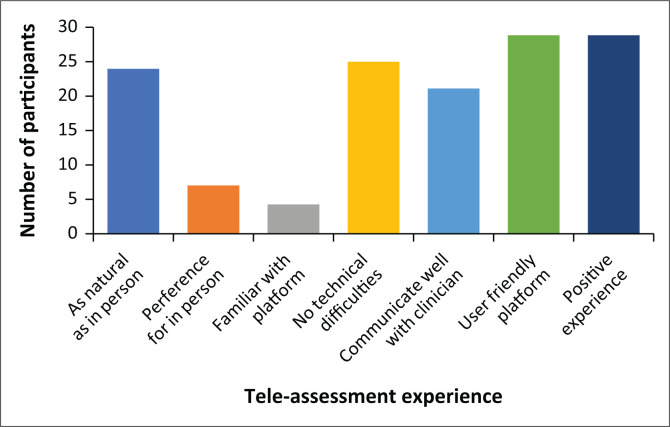
Caregivers’ experiences of the tele-assessment format.

Most caregivers also experienced no technical difficulties – such as poor connectivity or delayed video feedback with only 16.7% (*n* = 5 out of 30) of the participants reporting technical difficulties. Difficulties were largely attributed to poor internet connectivity and addressed easily, for example, by switching from audio and video call to audio-only call. A majority of the participants reported being able to communicate well with the clinician (70%, *n* = 21 out of 30). Many participants experienced the platform used during the tele-assessment (Google Meet™) as user friendly (96.7%, *n* = 29 out of 30), while only one participant reported feeling neutral about the platform (3.3.%, *n* = 1 out of 30). Overall, the majority (96.7%, *n* = 29 out of 30) of the caregivers reported their experience with tele-assessment as positive, while one caregiver (3.3%, *n* = 1 out of 30) reported their experience as neutral (Table 3 and Figure 1).

**TABLE 3:** Verbatim responses to the question: Would you consider tele-assessment as a service you might use again in future?

Participant number	Responses
P5	‘It’s efficient, safe [*with regards to COVID-19*] and very useful during a lockdown.’
P11	‘It’s the way of the future. Wouldn’t use it for building long-term relationships but otherwise it works fine.’
P14	‘More convenient than travelling somewhere. If I can see the person and hear them clearly, no need for a physical meeting in my opinion.’
P16	‘Convenient for adults maybe.’
P26	‘I enjoy personal contact. However it sometimes is more practical to do virtual sessions.’
P26	‘User friendly and informative. Was able to discuss my son’s development thoroughly without feeling I had a time constraint.’

### Feasibility and applicability for future use

A large number of the participants considered tele-assessment as something they would use again in future (90.0%, *n* = 27 out of 30). When asked to elaborate on their answer, 24 participants completed the open-ended question and stated that they found tele-assessment to be practical and informative (20.8%, *n* = 5 out of 24), convenient and resource-saving (54.2%, *n* = 13 out of 24) and safe with regard to the COVID-19 pandemic (12.5%, *n* = 3 out of 24). No caregivers reported that they would not use tele-assessment again; however, some participants felt neutral about it (10.0%, *n* = 3 out of 30) and felt unsure about the format when asked to elaborate (12.5%, *n* = 3 out of 24). In an open-ended question concerning what the caregivers would change about the tele-assessment format, most caregivers reported that no changes were necessary (76.2%, *n* = 16 out of 21) – however, 9.5% (*n* = 2 out of 21) suggested the assessment questions be sent before the tele-assessment session took place. Another participant (4.8%, *n* = 1 out of 21) reported a preference for not being recorded.

**FIGURE 2:**
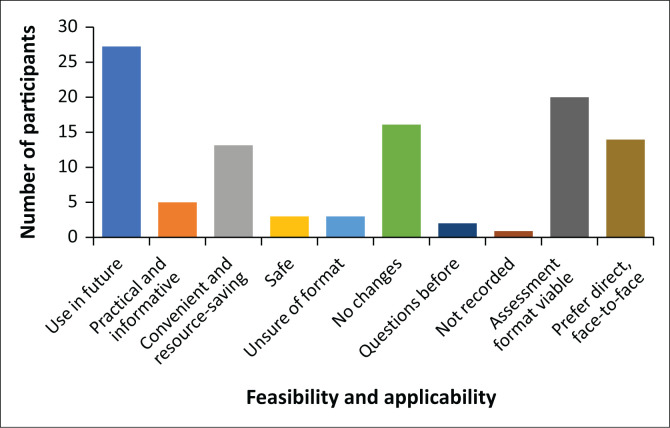
Feasibility and applicability for future use.

Regarding the participants’ perspectives of whether they felt tele-assessment was viable, two-thirds (66.7%, *n* = 20 out of 30) felt that the assessment format was viable, while 26.7% (*n* = 8 out of 30) reported feeling neutral about it with the remaining 6.7% (*n* = 2 out of 30) feeling that it was not viable (Table 4). The factors influencing viability, according to caregiver feedback, included: (1) preference for direct assessment of the child (53.8%, *n* = 14 out of 26); (2) confidence regarding their ability to report on their child’s development (30.8%, *n* = 8 out of 26) and (3) tele-assessment is convenient and saves resources, for example, transport (15.4%, *n* = 4 out of 26). It is notable that while the majority of participants reported perceiving tele-assessment as viable, when asked to elaborate on their answer, of the 26 participants who completed the open-ended question, 53.8% (*n* = 14 out of 26) indicated that they still preferred direct, face-to-face developmental assessment of their children (Figure 2). Although the child was able to be physically present throughout the interview, the nature of the Vineland-3 did not involve direct elicitation of any behaviours from the child.

**TABLE 4:** Verbatim responses to the question: Do you believe tele-assessment is a viable format for the developmental assessment of children from birth to 36 months?

Participant number	Responses
P1	‘There were some slight delays and it was sometimes more difficult to interpret subtle social cues’.
P2	‘Where caregivers are involved but probably better to evaluating the child in person.’
P13	‘The caregiver is the one who spends the most time with the child and is best equipped to answer questions about their development. There is no need for the professional to physically assess the child if the caregiver can give thorough feedback on any questions.’
P23	‘The only portion I will propose is perhaps to bring the child into the conversation depending on what is evaluated. I do believe it could be beneficial to hear the child or see the child doing activities or even using video to supplement and support observation made by the caregiver.’

From the open-ended questions, which were answered by 25 respondents, 60% (*n* = 15 out of 25) indicated the child was the firstborn and 40% (*n* = 10 out of 25) indicated that the child was not the firstborn. When the child that was the subject of the developmental assessment was not the firstborn, there was a significant agreement (*x*^2^ = 6.303, *p* = 0.035) that tele-assessment was user-friendly, informative and practical, in comparison to respondents where the child is the firstborn (13.3%, *n* = 2 out of 15). Respondents discussing firstborn children, however, significantly agreed (*x*^2^ = 6.303, *p* = 0.035) that the format was overall convenient and saved on transport costs more so than respondents where their children were not the firstborns (40.0%, *n* = 4 out of 10).

## Discussion

Tele-assessment is developing as a solution to ongoing barriers to accessing services, particularly during the COVID-19 pandemic (Farmer et al., 2020). Several caregivers who participated in this study reported feeling tele-assessment to be a safer alternative with regards to the pandemic (12.5%, *n* = 3 out of 24). While the majority of caregivers indicated a preference for more direct, face-to-face assessment (53.8%, *n* = 14 out of 26) or a hybrid approach, this points towards tele-assessment being a relatively newer concept to families in South Africa (Mathews et al., 2019). Additionally, this preference may indicate that caregivers could be concerned about the clinician’s established rapport with the family or lack thereof. A hybrid means of tele-assessment may thus be considered – such as the combination of supplementary video material to inform the tele-assessment, in order to assist with the development of a therapeutic alliance.

The implementation of the PEDS tools in the PHC context appears to be the only developmental tele-practice currently being investigated in South Africa (Du Toit et al., 2021; Maleka et al., 2016; Van der Merwe et al., 2019). Additionally, the PEDS is a screening tool and not a comprehensive developmental assessment such as the Vineland-3, which was utilised in the current study. The use of comprehensive developmental assessments in tele-assessment requires further investigation in the South African context.

Because of the novelty of this study, several limitations were present. The sample was small (*n* = 30) and homogenous (100% mothers, 86.7% of one demographic representation of middle- to high-income class) and thus was not representative of South Africa as a whole. Future research needs to be conducted with a larger sample, with groups of participants from a variety of economic statuses and demographic backgrounds to present findings that are more representative of South Africa and other LMICs. The homogeneity of the participants limits the applicability of the results to other populations. This study was also limited in that it had no comparison group, and no direct face-to-face assessment was conducted, thus resulting in no traditional standard to which the results could be compared to and validated. Future studies should not only focus on tele-assessment provided as a solution in LMICs but also on a global scale and with different permutations of the tele-assessment format to allow for more generalisability of results.

The participants of the current study identified tele-assessment as a viable solution, which therefore needs a greater supporting body of research. This study is the first to report on caregiver perspectives of interview-based early developmental tele-assessment, in the South African context. Furthermore, this study reports that tele-assessment was well received among caregivers of children from birth to 36 months, indicating that it has promise to be implemented on a larger scale in the realm of early developmental assessment and as a solution to overcoming barriers to accessing developmental services. The South African National Department of Health has identified telehealth as a crucial means for the provision of healthcare services, particularly when considerations around physical and social distancing are paramount because of challenging circumstances frequently arising. This approach is deemed especially significant for facilitating access to healthcare in underserved South African communities (South African National Department of Health, 2012).

### Clinical implications

This study was able to provide insight on the use of caregiver-report through means of tele-assessment and caregivers’ perceptions thereof.

#### The use of tele-assessment during challenging circumstances

Caregivers found tele-assessment to be practical and informative, as well as a safer alternative and a viable solution during challenging circumstances such as the COVID-19 pandemic, indicating that it has promise to be implemented on a larger scale in the realm of early developmental assessment and as a solution to overcoming barriers that hinder access to developmental care. Following the COVID-19 pandemic, telehealth expanded as a method of service delivery within South Africa, with expectations for its continued growth, particularly in light of the deployment of complimentary public Wi-Fi networks by municipal authorities, including Tshwane’s TshWi-Fi initiative. This increase underscores the necessity for evidence supporting the efficacy of telehealth in LMICs, like South Africa. This study offered the opportunity for developmental monitoring in the midst of a strict nationwide lockdown period in response to the COVID-19 outbreak, which meant that caregivers did not have to worry about exposing their child to COVID-19 while still receiving early childhood development (ECD) services. It is clear that caregivers’ concerns about their children’s safety affect their preferred method of receiving ECD services, and tele-assessment provides a valuable alternative in that regard. This furthermore implies that the use of tele-assessment for caregiver interviews can be considered for consistent service delivery by clinicians, especially within the context of an ongoing global crisis such as COVID-19. As only the caregiver and the clinician need to be present and no physical contact is needed, neither the caregiver, child nor the clinician are exposed to the risk of COVID-19. Tele-assessment also overcomes barriers to developmental assessment such as poor follow-up adherence because of physical challenges and distance. The researcher was able to assess children who resided in separate provinces with ease.

#### Use of a hybrid tele-assessment model for developmental monitoring

**FIGURE 3:**
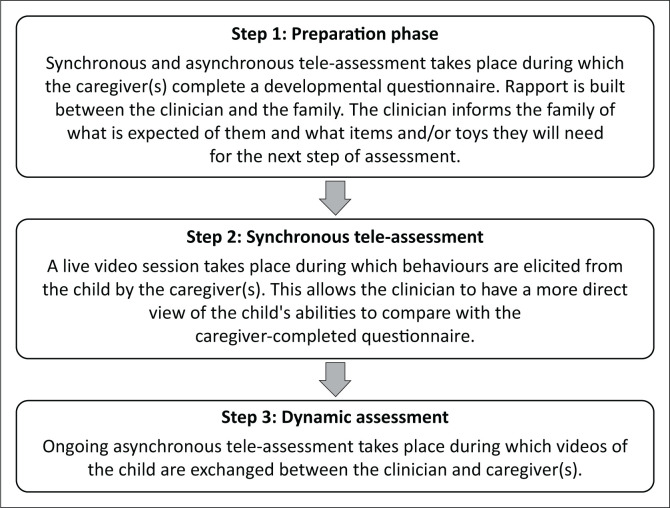
A proposed hybrid tele-assessment model.

Many caregivers reported to prefer direct, face-to-face assessment (53.8%, *n* = 14 out of 26), and it is suggested to do this in conjunction with tele-assessment where possible. A potential hybrid method of tele-assessment that serves to uphold the principles of quality telepractice and caters to each family’s needs is proposed (Figure 3). It is recommended that, first, a period of synchronous and asynchronous tele-assessment takes place during which rapport is established and questions for the developmental assessment are forwarded to the caregivers. This would be done synchronously, for example, a telephonic conversation, and asynchronously, such as email or instant messaging. The Vineland-3 has a caregiver questionnaire, which consists of the same questions as the clinician-administered interview, presented in a way that caregivers rate their child’s development themselves. This will allow the clinician to gain caregivers’ perspectives on the child’s development, as well as help the caregivers feel more prepared for the next step in the tele-assessment process. During this step, the caregivers will also be informed of anything they may need for the next step, such as any toys or items necessary to elicit behaviours during the assessment. The proposed next step would be a synchronous video session that should be arranged by the clinician, caregivers and the child. During the appointment, the caregivers will elicit behaviours from the child for the purpose of the clinician assessing these behaviours to compare against the information gathered from the caregiver-completed questionnaire, to fill in any potential gaps regarding the child’s developmental profile. This will allow for a more holistic view of the child and their development, as well as to help the caregivers feel that their child is involved in the assessment process and feel assured that the clinician is not overlooking anything important regarding their child’s development. The final proposed stage of the tele-assessment process, should the child go on to receive intervention, is the use of asynchronous video exchange for ongoing monitoring to supplement tele-intervention. This type of dynamic assessment is a vital component of the intervention process, as it not only maintains the relationship between clinician and family but also provides an important view of the child’s ongoing development and progress (Boers et al., 2013).

## Conclusion

Caregivers are the key stakeholders in the developmental monitoring of children, making it vital to consider their perspectives, concerns and priorities in the research of early developmental service delivery approaches. This study was able to provide insight on the use of interview-based early developmental tele-assessment and caregivers’ perceptions thereof. The overall perceived experiences were positive, and tele-assessment appears to be a viable approach for caregivers to access developmental care services during circumstances such as COVID-19. The application of caregiver-report through tele-assessment requires further investigation to generalise the results to other populations.
